# Taking the Case*Read before the Kansas State Homœopathic Medical Society.

**Published:** 1895-08

**Authors:** J. A. Tomhagen

**Affiliations:** Chicago, Ill.


### TAKING THE CASE.*

J. A. Tomhagen, M. D., Chicago, Ill.

Having been asked to contribute a paper for your bureau, I offer this as embracing some of those vital questions always of interest to the earnest and busy physician. Hahnemann said, "A case well taken is half cured." I have found that my success has been proportionate to the care exercised in making the first examination. Most of our failures are directly traceable to overlooking the individual *peculiarities* as fixed in each and every patient, as well as the peculiar complexes of symptoms which are wont to appear from time to time at regular intervals, and, indeed, in orderly succession, at times from within outward, and from above downward and in reverse order—the former salutary, the latter detrimental to the patient's well-being. Eruption of cutaneous diseases is invariably followed by diminution of internal distress. Suppression of skin diseases means aggravation of internal ailments or the development of the same, if not existing prior to the eruption. Disease passing from head to feet is always followed by a cure of more vital centres. When ailments travel from below upward serious trouble may be confidently predicted.

This orderly crescendo and decrescendo of phenomena must be appreciated by the physician ere he can hope to get a purchase

---

*Read before the Kansas State Homœopathic Medical Society.

upon the latent principles embodied in the law of *similia.* These vital principles must be perceived and conceived before they can evolve an objective entity, so to speak, and become a constant and ever-present fact in the mind of the physician. Let him once grasp the eternal verity of this fixed law, then nothing that man might contrive, however extensively lauded (Koch's lymph, Brown-Sequard's Elixir, *et al.*), can ingratiate itself to dethrone those agents which have been thoroughly proven and tested and not found wanting.

One of the first rules of practice in regard to *taking a case,* and one I never deviate from, since it was inculcated by the masters, is the manner of eliciting symptoms. Shakespeare said, "By indirections, find directions out." It requires great skill and tact to so evoke the symptoms as not to prejudice or bias the patient in his or her statements. Avoid interruptions and questions that can be answered by yes or no. Oftentimes they expatiate with wonderful volubility concerning their condition, and unwittingly divulge many noteworthy facts before the physician is prepared to write their statements. Again, their utterances are monosyllabic—"yes" or "no." It is in this latter class that after a few pertinent questions with negative results a dose of Placebo serves as an entering wedge which causes the tongue to loosen because the patient feels that you have found his trouble and understand his case thoroughly, and therefore your speedy prescription. He at once throws off the restraint and feels perfectly at ease, and proceeds, oftentimes, to unburden the mind thus: "There is one thing I forgot to mention, and it seems very strange, this terrible pain begins every morning at three o'clock. I don't know why it should, but it does." This aggravation decided in favor of Thuja, which cured the case. Ten successive nights this pain, a violent prosopalgia, recurred at precisely three A. M., in spite of Morphine and Quinine. One dose of Thuja cured the case. This symptom might easily have escaped one at the time, because of the reticent mood of the patient. The dose of Placebo relaxed the patient, not because of its medicinal qualities but because he felt that I had found his remedy, and the inner consciousness being at ease, he gave expression to this *peculiar fact.* Often a patient turns on his heels, as he is about to vanish through the door, and mentions some very important symptom, one that would have decided in favor of another remedy, but he had simply received Placebo to await further developments; but now the remedy stands out in *alto relievo*, and can be given with perfect confidence and positive assurance.

This manner of proceeding enables us to observe the *peculiar subjective expressions* or *symptoms,* often alone sufficient, upon which to base a prescription, without further questioning. When, however, the subjective image is obscure, objective phenomena are generally present that will throw some light on past experiences. An enlarged and indurated occipital gland, maculæ patches and crippled nails. A poorly developed chin means a congenitally weak heart. Lack of fullness in the lower part of the face signifies weak digestion. Narrowness between the malar bones indicates a tendency to pulmonary troubles. These facts speak volumes to the initiated, and indicate the proper line of interrogations. Many more might be adduced but these suffice for the present to show how interesting and instructive this study might become.

While the patient is adjusting himself physically and mentally, and endeavoring to decide where to begin with the recital of his symptoms, we should, inadvertently, proceed to take his measure, "size him up," as it were. This means a great deal more than many even suspect. Hahnemann, Bœnninghausen, and Jahr laid great stress upon studying *human nature.* In truth I have found that much depends upon the accuracy with which this is done. Those having a vivid, intuitive perception of human nature, rapidly detect its shortcomings, upon both the physical and mental planes.

Hahnemann said, "Disease is a distunement of the vital force." I accept this, in toto. To distune the vital force means to throw it out of harmony. An instrument to be distuned or out of harmony, means that discordant sounds will result, and this implies too rapid *vibrations* of some chords and too slow of others. So when the *vital force* is out of tune, it must follow that some of its chords vibrate too fast and others not fast enough, resulting in what is called *dis-ease*. The animal, mineral, and vegetable kingdoms furnish *substances* that have *specific* affinities for various parts and sections of man, distuning these in greater or less degree, resulting in phenomena or effects which we call symptoms. The process by which these are obtained is called a proving, which should be made upon a healthy individual, and one for whom the element has a *special affinity*, thus enabling it to bring out its most *peculiar features*. For example, Belladonna has most affinity with plethoric individuals; those in whom the *arterial system* predominates over the *venous*. These people are endowed with perfect vitality, are full and rotund, the middle section of the brain is large and active. They are full of vivacity and expression, the result of an active arterial circulation. The delirium is active, the mania wild, the pain acute and violent, of an intermittent character, and characteristically worse in the afternoon at three P. M., when the arterial excitement is at its height. Acon., Amyl-nitrite, and Glon. are quite similar to Bell. in their affinity for full-blooded active individuals, and therefore especially adapted to the young. Old people would rarely require these remedies as a rule, since their life forces are waning. Gels., unlike Acon., Bell., Nitro-glyc., produces venous plethora, and therefore all of its symptoms partake of a depressing character, the pulse is full but slow and soft, not bounding like Acon. and Bell. Great restlessness, thirst, and anxiety characterize Acon., while Gels. is less restless, thirstless, and indifferent. Wants to be let alone. Bell. causes thirst for a little and often in a characteristic way. Again, Gels. has a specific affinity for *tall, bilious, nervous* individuals, in whom the nervous congestion causes temporary suspension of innervation in the nerve centres and consequent depression of the entire nervous system, giving the great characteristic of *prostration* both mental and physical. At a glance the active practitioner can detect these *peculiar* and *innate tendencies* in each individual patient, by familiarizing himself with these underlying principles.

He must study *human nature* as Hahnemann taught. The ability to detect *peculiar symptoms* is facilitated by this method. In a paper of this kind we can simply indicate a few of the peculiar conditions necessary for the selection of the remedy. In pneumonia, for example, where the choice lies between Kali-bichrom. and Sang. it is oftentimes quite difficult to decide which to prescribe at a certain stage when the *thick stringy yellowish white* mucus is a prominent factor. If we remember, however, that Kali-bichrom. has a special affinity for corpulent light-haired persons, and Sang. for heavy set, bilious individuals with dark hair and eyes, the choice becomes comparatively easy, providing two, three, or four other peculiar and differentiating symptoms are not wanting. Some will say: "I have had excellent results from Puls. in brunettes, and Nux in blondes." I don't dispute this in the least. Some say: "I prefer the CM and millionth potencies, and have had satisfactory results therefrom." Another uses the lower attenuations and is equally satisfied with his results through years of experience. Old-school physicians will tell you that they have good results from their remedies. Some alternate two remedies or give three or more in rotation, and are content to continue in this method, claiming good results. I happen to know that some prescribe Puls. and Nux regardless of *individual peculiarities*, having given as many as eight remedies in one month, on an average two per week, blindly administering this and that medicament for transitory and ephemeral symptoms, and are as far from a cure at the end of six months as they were in the beginning. Good results may satisfy some, but I want the *best* results that can be had in the shortest time, *in curable cases.* Having decided *the remedy*, the next question which naturally suggests itself is, How shall we administer it? My experience has been exclusively with potencies ranging between the 200th and CM, occasionally even higher, formerly giving them in solution, a spoonful every two hours, till a decided change was noticeable, and four or five powders dry on the tongue, a powder every two hours in chronic affections. Experience has taught me that a dose dry on the tongue is sufficient for any curable case, and likewise enough as a palliative in incurable ones, wonderful euthanasia being the result in moribund cases, far surpassing the transitory and simply depressing effects of Morphia and the bromides. I do not doubt in the least the beneficial effects of remedies in potencies lower than the 200th. Hahnemann and his disciples made remarkable cures with potencies ranging lower, and many are doing the same at this day. *The chief question is the proper affiliation of the remedy in each individual case. The closer the affinity, the more danger in prescribing crude vegetable and animal drugs; or, in other words, the more violent the aggravation from the lower potencies.* Therefore the *most accurate* prescribers found the most beneficial effects from the higher potencies. They are more penetrating because of their *high rate of vibration.* This explanation satisfies me, but I will not quarrel with any one who may choose to differ; it is simply my opinion. If any one has a better and more scientific explanation I am willing to accept it. The fact nevertheless remains the same, that these potencies *act.* The next important question is in regard to repetition of dose. I never repeat as long as the patient continues to improve. If the patient continues to grow worse after the first prescription, the medicine was improperly selected, and another is in order after twenty-four hours, because the conditions invariably change from day to day, and often from hour to hour. It is oftentimes difficult to decide whether the remedy given has aggravated the patient's condition, or whether the disease is progressing in its course unimpeded.

The only safe guide I know of is this: Is the apparent aggravation in harmony with the action of the medicine? If so, I let it alone, if not, I endeavor to adjust another remedy, either compatible with or complementary to it. An incompatible remedy would spoil the prospect of a speedy recovery. Should the patient after the lapse of twenty-four hours evince symptoms similar to those found at the first visit, a second dose would be in order, but this is *rarely* necessary; generally nothing is required and the patient goes on to apparent recovery.

I say *apparent* because there is usually something constitutionally wrong in these patients that require systematic treatment. The remedies selected from the vegetable kingdom are generally best adapted to, or most appropriate for acute diseases, and should some chronic miasm come to the surface after the subsidence of the acute ailment, one of the mineral elements is likely to come into requisition to complete the cure, as Calc-c. after Bell., Sulph. after Acon., Alumina after Bry., Sil. after Puls., Natr-m. after Ign., Stan. after Puls., Ant-tart. after Ipec., Iod. after Lyco., etc. Some of these remedies, though belonging to the vegetable kingdom, as Lyco. and Puls., will carry a case along for weeks with unremitting improvement.

Now as to repetition of medicine in chronic diseases. These chronic ailments exist because of some *peculiar bias* of the individual from the very hour of his birth. He is crippled or dwarfed as to some part of his system in the beginning. This becomes more and more manifest as the years roll on. Improper balance between the arterial and venous circulation, sluggish liver with its host of consequences: hemorrhoids, constipation, kidney troubles, weak lungs or stomach; pancreas inactive; some organs of the brain deficient and others overactive, with corresponding manifestations. Many of these can be corrected by judicious medical care from the beginning. Sulph., Calc., Sil., Phos., Lyc., etc., properly administered at the outset of the child's earthly career, will cause it to unfold physiologically by directing the various functions in the way they should go.

Most of these inveterate ailments are intensified by improper modes of life which should be corrected as far as practicable and then one of anti-psoric, anti-sycotic, or anti-syphilitic action properly affiliated. The management of chronic troubles *per se* is so *intricate* that it is impossible to do it justice at the conclusion of this paper. I might add, however, that the *totality* of *peculiar subjective* and *objective phenomena, signs* and *symptoms* of each individual case, are our only guide in the selection of remedies for chronic diseases. Trusting that I have brought out a few facts that others might corroborate, I submit them for your discussion.

